# Predictors of clinically significant quality of life impairment in Parkinson’s disease

**DOI:** 10.1038/s41531-021-00256-w

**Published:** 2021-12-16

**Authors:** Diego Santos García, Teresa de Deus Fonticoba, Carlos Cores, Guillermo Muñoz, Jose M. Paz González, Cristina Martínez Miró, Ester Suárez, Silvia Jesús, Miquel Aguilar, Pau Pastor, Lluis Planellas, Marina Cosgaya, Juan García Caldentey, Nuria Caballol, Inés Legarda, Jorge Hernández Vara, Iria Cabo, Luis López Manzanares, Isabel González Aramburu, María A. Ávila Rivera, Maria J. Catalán, Víctor Nogueira, Víctor Puente, María Ruíz de Arcos, Carmen Borrué, Berta Solano Vila, María Álvarez Sauco, Lydia Vela, Sonia Escalante, Esther Cubo, Francisco Carrillo Padilla, Juan C. Martínez Castrillo, Pilar Sánchez Alonso, Maria G. Alonso Losada, Nuria López Ariztegui, Itziar Gastón, Pedro Clavero, Jaime Kulisevsky, Marta Blázquez Estrada, Manuel Seijo, Javier Rúiz Martínez, Caridad Valero, Mónica Kurtis, Oriol de Fábregues, Jessica González Ardura, Carlos Ordás, Luis M. López Díaz, Darrian McAfee, Pablo Martinez-Martin, Pablo Mir, COPPADIS Study Group, Daniela A. Adarmes, Marta Almeria, Araceli Alonso Cánovas, F. Alonso Frech, Ruben Alonso Redondo, Ignacio Álvarez, Ángel Aneiros Díaz, Sandra Arnáiz, Sonia Arribas, Arancha Ascunce Vidondo, Noemí Bernardo Lambrich, Helana Bejr-Kasem, María A. Botí, María T. Buongiorno, Carolina Cabello González, A. Cámara Lorenzo, Héctor Canfield Medina, Fátima Carrillo, Elena Casas, Ana Cortina Fernández, Anna Cots Foraster, Ane Crespo Cuevas, Mónica Díez-Fairen, Julio Dotor García-Soto, Elena Erro, Elena Estelrich Peyret, Noelia Fernández Guillán, Pedro Gámez, Mercedes Gallego, Cristina García Campos, Jose M. García Moreno, María P. Gómez Garre, Vívtor Gómez Mayordomo, Javier González Aloy, Beatriz González García, María J. González Palmás, González Toledo, R. Gabriel, Ana Golpe Díaz, Mireia Grau Solá, Gemma Guardia, Andrea Horta-Barba, Daniel Idoate Calderón, Jon Infante, Carmen Labandeira, Miguel A. Labrador, Francisco Lacruz, Melva Lage Castro, Sonia Lastres Gómez, Balbino López Seoane, Sara Lucas del Pozo, Yolanda Macías, Marina Mata, Gloria Martí Andres, María J. Martí, Maria T. Meitín, Manuel Menéndez González, Carlota Méndez del Barrio, Javier Miranda Santiago, Morales Casado, I. María, Antonio Moreno Diéguez, Alba Novo Amado, Sabela Novo Ponte, Javier Pagonabarraga, Isabel Pareés, Berta Pascual-Sedano, Aída Pérez Fuertes, Rafael Pérez Noguera, Ana Planas-Ballvé, Marian A. Prats, Cristina Prieto Jurczynska, Mercedes Pueyo Morlans, Arnayu Puig Daví, Nuria Redondo Rafales, Luisa Rodríguez Méndez, Amparo B. Rodríguez Pérez, Florinda Roldán, Macarena Sánchez-Carpintero, Gemma Sánchez Díez, Antonio Sánchez Rodríguez, Pilar Santacruz, José C. Segundo Rodríguez, María Sierra Peña, Juan P. Tartari, Laura Vargas, Clara Villanueva, Bárbara Vives, María D. Villar

**Affiliations:** 1 CHUAC, Complejo Hospitalario Universitario de A Coruña, A Coruña, Spain; 2 CHUF, Complejo Hospitalario Universitario de Ferrol, A Coruña, Spain; 3 Unidad de Trastornos del Movimiento, Servicio de Neurología y Neurofisiología Clínica, Instituto de Biomedicina de Sevilla, Hospital Universitario Virgen del Rocío/CSIC/Universidad de Sevilla, Seville, Spain; 4 CIBERNED (Centro de Investigación Biomédica en Red sobre Enfermedades Neurodegenerativas), Álava, Spain; 5 Hospital Universitari Mutua de Terrassa, Terrassa, Barcelona, Spain; 6 Neurología, Clínica del Pilar, Barcelona, Spain; 7 Hospital Clínic de Barcelona, Barcelona, Spain; 8 Centro Neurológico Oms 42, Palma de Mallorca, Spain; 9 Consorci Sanitari Integral, Hospital Moisés Broggi, Sant Joan Despí, Barcelona, Spain; 10 Hospital Universitario Son Espases, Palma de Mallorca, Spain; 11 Hospital Universitario Vall d´Hebron, Barcelona, Spain; 12 Complejo Hospitalario Universitario de Pontevedra (CHOP), Pontevedra, Spain; 13 Hospital Universitario La Princesa, Madrid, Spain; 14 Hospital Universitario Marqués de Valdecilla, Santander, Spain; 15 Consorci Sanitari Integral, Hospital General de L´Hospitalet, L´Hospitalet de Llobregat, Barcelona, Spain; 16 Hospital Universitario Clínico San Carlos, Madrid, Spain; 17 Hospital Da Costa, Burela Lugo, Spain; 18 Hospital del Mar, Barcelona, Spain; 19 Hospital Universitario Virgen Macarena, Sevilla, Spain; 20 Hospital Infanta Sofía, Madrid, Spain; 21 Institut d’Assistència Sanitària (IAS) - Institut Català de la Salut, Girona, Spain; 22 Hospital General Universitario de Elche, Elche, Spain; 23 Fundación Hospital de Alcorcón, Madrid, Spain; 24 Hospital de Tortosa Verge de la Cinta (HTVC), Tortosa Tarragona, Spain; 25 Complejo Asistencial Universitario de Burgos, Burgos, Spain; 26 Hospital Universitario de Canarias, San Cristóbal de la Laguna, Santa Cruz de Tenerife, Spain; 27 Hospital Universitario Ramón y Cajal, IRYCIS, Madrid, Spain; 28 Hospital Universitario Puerta de Hierro, Madrid, Spain; 29 Hospital Álvaro Cunqueiro, Complejo Hospitalario Universitario de Vigo (CHUVI), Vigo, Spain; 30 Complejo Hospitalario de Toledo, Toledo, Spain; 31 Complejo Hospitalario de Navarra, Pamplona, Spain; 32 Hospital de Sant Pau, Barcelona, Spain; 33 Hospital Universitario Central de Asturias, Oviedo, Spain; 34 Hospital Universitario Donostia, San Sebastián, Spain; 35 Hospital Arnau de Vilanova, Valencia, Spain; 36 Hospital Ruber Internacional, Madrid, Spain; 37 Hospital Universitario de Cabueñes, Gijón, Spain; 38 Hospital Rey Juan Carlos, Madrid, Spain Madrid, Spain; 39 Complejo Hospitalario Universitario de Orense (CHUO), Orense, Spain; 40 Univeristy of Pennsylvania, Pennsylvania, USA; 41 Hospital Universitario Lucus Augusti (HULA), Lugo, Spain; 42 Hospital General Juan Cardona, Ferrol A Coruña, Spain; 43 Clínica Universidad de Navarra, Pamplona, Spain

**Keywords:** Parkinson's disease, Quality of life

## Abstract

Quality of life (QOL) plays an important role in independent living in Parkinson’s disease (PD) patients, being crucial to know what factors impact QoL throughout the course of the disease. Here we identified predictors of QoL impairment in PD patients from a Spanish cohort. PD patients recruited from 35 centers of Spain from the COPPADIS cohort from January 2016, to November 2017, were followed up during 2 years. Health-related QoL (HRQoL) and global QoL (GQoL) were assessed with the 39-item Parkinson’s disease Questionnaire (PDQ-39) and the EUROHIS-QOL 8-item index (EUROHIS-QOL8), respectively, at baseline (V0) and at 24 months ± 1 month (V2). Clinically significant QoL impairment was defined as presenting an increase (PDQ-39SI) or decrement (EUROHIS-QOL8) at V2 ≥ 10% of the score at baseline (V0). A comparison with a control group was conducted for GQoL. GQoL did not change significantly in PD patients (*N* = 507; *p* = 0.686) or in the control group (*N* = 119; *p* = 0.192). The mean PDQ-39SI was significantly increased in PD patients (62.7 ± 8.5 years old; 58.8% males; *N* = 500) by 21.6% (from 16.7 ± 13 to 20.3 ± 16.4; *p* < 0.0001) at V2. Ninety-three patients (18.6%) presented a clinically significant HRQoL impairment at V2. To be younger (OR = 0.896; 95% CI 0.829–0.968; *p* = 0.006), to be a female (OR = 4.181; 95% CI 1.422–12.290; *p* = 0.009), and to have a greater increase in BDI-II (Beck Depression Inventory-II) (OR = 1.139; 95% CI 1.053–1.231; *p* = 0.001) and NMSS (Non-Motor Symptoms Scale) (OR = 1.052; 95% CI 1.027–1.113; *p* < 0.0001) total scores from V0 to V2 were associated with clinically significant HRQoL impairment at the 2-year follow-up (Hosmer–Lemeshow test, *p* = 0.665; *R* ^2^ = 0.655). An increase in ≥5 and ≥10 points of BDI-II and NMSS total score at V2 multiplied the probability of presenting clinically significant HRQoL impairment by 5 (OR = 5.453; 95% CI 1.663–17.876; *p* = 0.005) and 8 (OR = 8.217; 95% CI, 2.975–22.696; *p* = 0.002), respectively. In conclusion, age, gender, mood, and non-motor impairment were associated with clinically significant HRQoL impairment after the 2-year follow-up in PD patients.

## Introduction

Parkinson’s disease (PD) is a complex disorder in which different motor and non-motor symptoms (NMS) can be present with a frequency and severity that varies among patients over time^1^. Both motor and NMS are important because they negatively impact the patient’s quality of life (QoL). Different studies have analyzed what factors contribute to a poor QoL in PD patients^2–13^. Recently, we observed that NMS burden, mood, and gait problems were the most relevant factors affecting health-related (HRQoL) and global perceived QoL (GQoL) in non-demented PD patients from the Spanish cohort COPPADIS^14^. These results aligned with other cross-sectional studies observations^15–17^. However, with regard to how the QoL of PD changes throughout the course of the disease, there is much less information^18–23^ and prospective longitudinal studies are needed. In clinical practice, it is important to know what factors worsen PD patients’ QoL with the intention to carry out effective interventions. Known information limited by factors from the studies such as the sample size, the differences between scales used for assessing QoL, the different types of QoL assessed, being a non-multicenter study, the absence of a control group, and/or the lack of a global evaluation including different aspects that could impact on QoL^18–23^. In addition, the impact of some complications on QoL in advanced PD has been analyzed before^24,25^. However, it is not clear what the significance of short-term changes in QoL is in early PD patients or what factors contribute to it when an extensive assessment considering motor and NMS is performed^26^. It is remarkable that NMS occur not only in advanced but also in the early stages of PD. Some symptoms, for example, olfactory deficit, constipation, rapid-eye-movement sleep behavior disorder, and depression, can even precede the appearance of motor symptoms by many years^1^. By the contrary, others such as psychosis or dementia are not present. The first years are conditioned by the acceptance of the diagnosis, but in general, the patient has greater autonomy. In this context, it is essential to know what influences the changes in the PD patient QoL perception with the intention of being able to act as soon as possible.

The aim of the present study was to (1) analyze the change in HRQoL and GQoL in PD patients from the COPPADIS cohort after the 2-year follow-up, (2) to compare with a control group, and (3) to identify predictors of clinically significant QoL impairment in the PD group. Finally, a subanalysis was conducted in a subgroup of patients with early PD (≤5 years of disease duration).

## Results

### Changes in assessments from V0 to V2

**Fig. 1:**
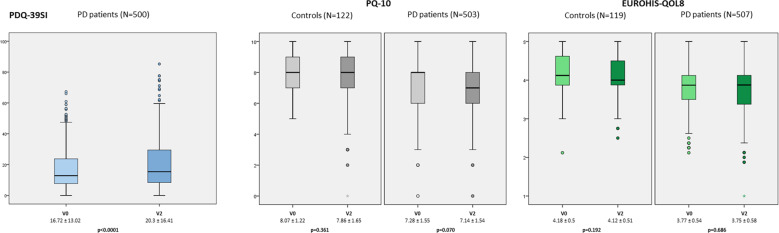
Change in PDQ-39SI, PQ-10, and EUROHIS-QOL8 scores from V0 (baseline) to V2 (2 year ± 1 month) in PD patients and/or controls. Data are presented as box plots, with the box representing the median and the two middle quartiles (25–75%). *p*-values were computed using the Wilcoxon-signed rank test. Mild outliers (O) are data points that are more extreme than Q1 − 1.5 * IQR or Q3 + 1.5 * IQR. EUROHIS-QOL8, European Health Interview Survey-Quality of Life 8-Item Index; PDQ-39SI, 39-item Parkinson’s Disease Quality of Life Questionnaire Summary Index.

After the 2-year follow-up, GQoL did not change significantly in PD patients (from PQ-10_V0_ of 7.28 ± 1.55 to PQ-10_V2_ of 7.14 ± 1.54 [*N* = 503; *p* = 0.070]; from EUROHIS-QOL8_V0_ of 3.77 ± 0.54 to EUROHIS-QOL8_V2_ of 3.75 ± 0.58 [*N* = 507; *p* = 0.686]) or in the control group (from PQ-10_V0_ of 8.07 ± 1.22 to PQ-10_V2_ of 7.86 ± 1.65 [*N* = 122; *p* = 0.361]; from EUROHIS-QOL8_V0_ of 4.18 ± 0.5 to EUROHIS-QOL8_V2_ of 4.12 ± 0.51 [*N* = 119; *p* = 0.192] (Fig. 1). The mean PDQ-39SI was significantly increased in PD patients (62.7 ± 8.5 years old; 58.8% males; *N* = 500) by 21.6% (from 16.72 ± 13.02 to 20.3 ± 16.41; *p* < 0.0001) at V2 (Table 1 and Fig. 1). By domains, the score of all domains of the PDQ-39SI at V2 was significantly higher than at V0 except for domain 4 (stigmatization) (Table 1). The change in the score of other scales from V0 to V2 in PD patients and controls is shown in Table 1.

**Table 1:** Changes in motor and non-motor symptoms, disability, and quality of life in PD patients and/or controls from V0 (baseline) to V2 (2 years ± 1 month).

	PD patients V0	PD patients V2	*p*_a_	Controls V0	Controls V2	*p*_b_
Hoehn & Yahr (OFF) (%)			**<0.0001**	N. A.	N. A.	N. A.
Stage 1	22.7	13.3				
Stage 2	68	77				
Stage 3–5	9.3	9.7				
UPDRS-III (OFF)	21.92 ± 10.53	25.26 ± 12.19	**<0.0001**	N. A.	N. A.	N. A.
UPDRS-IV	1.99 ± 2.41	2.65 ± 2.75	**<0.0001**	N. A.	N. A.	N. A.
FOGQ	3.76 ± 4.69	4.94 ± 5.18	**<0.0001**	N. A.	N. A.	N. A.
LEDD	577.48 ± 412.09	767.56 ± 307.1	**<0.0001**	N. A.	N. A.	
Number of non-antipark. drugs	2.35 ± 2.38	3.08 ± 2.65	**<0.0001**	2.04 ± .2.16	2.76 ± 2.35	**0.001**
PD-CRS	92 ± 15.65	90.26 ± 18.07	**<0.0001**	99.65 ± 13.56	99.68 ± 13.73	0.744
NMSS	45.08 ± 37.62	53.55 ± 42.28	**<0.0001**	14.74 ± 18.72	14.65 ± 21.82	0.428
BDI-II	8.28 ± 6.9	8.54 ± 7.48	0.472	4.56 ± 5.46	4.31 ± 5.5	0.776
PDSS	117.13 ± 24.48	117.85 ± 24.98	0.797	131.26 ± 17.41	126.67 ± 26.46	0.947
QUIP-RS	4.6 ± 8.8	4.66 ± 9.22	0.937	1.51 ± 3.73	1.32 ± 3.37	0.498
NPI	5.82 ± 7.88	6.17 ± 9.39	0.671	3.31 ± 7.15	2.64 ± 7.67	0.120
VAS-PAIN	2.61 ± 2.92	2.96 ± 2.88	**0.013**	1.49 ± 2.41	1.70 ± 2.32	0.319
VASF − physical	2.86 ± 2.67	3.17 ± 2.8	**0.010**	1.52 ± 2.35	1.29 ± 2.12	0.103
VASF − mental	2.09 ± 2.51	2.20 ± 2.61	0.538	1.29 ± 2.09	1.03 ± 1.97	0.273
ADLSL	88.58 ± 10.19	84.26 ± 13.38	**<0.0001**	98.87 ± 6.65	99.52 ± 2.15	0.285
PDQ-39SI	16.72 ± 13.02	20.3 ± 16.41	**<0.0001**	N. A.	N. A.	N. A.
Mobility	16.28 ± 19.2	21.31 ± 22.5	**<0.0001**			
Activities of daily living	17.83 ± 18.83	21.82 ± 21.37	**<0.0001**			
Emotional well-being	20.92 ± 19.52	23.53 ± 23.45	**<0.0001**			
Stigmatization	12.81 ± 19.24	14.14 ± 21.09	0.069			
Social support	7.29 ± 15.43	10.01 ± 19.09	**<0.0001**			
Cognition	18.51 ± 17.38	23.17 ± 20.16	**<0.0001**			
Communication	9.68 ± 14.44	13.55 ± 18.88	**<0.0001**			
Pain and discomfort	26.75 ± 22.33	28.67 ± 23.37	**0.009**			
PQ-10	7.28 ± 1.55	7.14 ± 1.54	0.070	8.07 ± 1.22	7.86 ± 1.65	0.361
EUROHIS-QOL8	3.77 ± 0.54	3.75 ± 0.58	0.686	4.18 ± 0.5	4.12 ± 0.51	0.192
Quality of life	3.8 ± 0.7	3.68 ± 0.67	**0.003**	4.14 ± 0.65	4.2 ± 0.63	0.298
Health status	3.18 ± 0.87	3.32 ± 0.93	**0.004**	3.97 ± 0.75	3.87 ± 0.82	0.148
Energy	3.76 ± 0.79	3.72 ± 0.86	0.266	4.15 ± 0.68	4.11 ± 0.69	0.531
Autonomy for ADL	3.61 ± 0.86	3.63 ± 0.88	0.852	4.24 ± 0.75	4.19 ± 0.61	0.983
Self-esteem	3.83 ± 0.76	3.82 ± 0.81	0.866	4.18 ± 0.68	4.00 ± 0.66	0.124
Social relationships	4.04 ± 0.67	3.94 ± 0.75	**0.004**	4.29 ± 0.65	4.19 ± 0.61	0.071
Economic capacity	3.84 ± 0.78	3.77 ± 0.8	0.091	4.07 ± 0.74	3.97 ± 0.81	0.078
Habitat	4.22 ± 0.67	4.21 ± 0.67	0.904	4.43 ± 0.63	4.29 ± 0.66	**0.016**

*p*-values were computed using the Wilcoxon-signed rank test or marginal homogeneity test. The results represent mean ± SD or %; *p*_a_, V2 vs V0 in PD patients; *p*_b_, V2 vs V0 in controls.

*ADLS* Schwab & England Activities of Daily Living Scale, *BDI-II* Beck Depression Inventory-II, *FOGQ* Freezing Of Gait Questionnaire, *LEDD* levodopa equivalent daily dose (mg), *NMSS* Non-Motor Symptoms Scale, *NPI* Neuropsychiatric Inventory, *PD-CRS* Parkinson’s Disease Cognitive Rating Scale, *PDSS* Parkinson’s Disease Sleep Scale, *QUIP-RS* Questionnaire for Impulsive-Compulsive Disorders in Parkinson’s Disease-Rating Scale, *UPDRS* Unified Parkinson’s Disease Rating Scale, *VAFS* Visual Analog Fatigue Scale, *VAS-Pain* Visual Analog Scale-Pain.

The bold values indicates statistically significant *p* values.

### Patients with vs without clinically HRQoL impairment

**Fig. 2:**
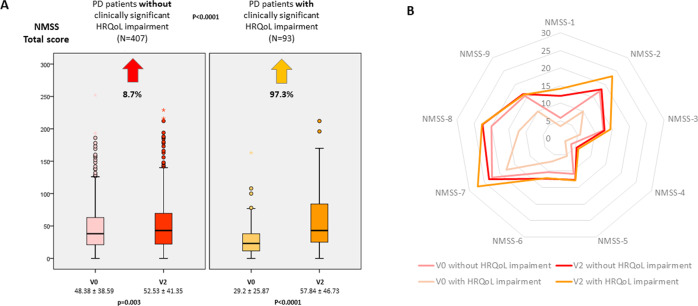
Evolution of NMS after 2-year follow up. **A** Change in the NMSS total score from V0 (baseline) to V2 (2 year ± 1 month) in PD patients without vs with clinically significant HRQoL impairment. **B** Mean score on each domain of the ESS scale at V0 and V2 in PD patients without vs with clinically significant HRQoL impairment. Data are presented as box plots, with the box representing the median and the two middle quartiles (25–75%). *p*-values were computed using the Wilcoxon-signed rank test. Mild outliers (O) are data points that are more extreme than Q1 − 1.5 * IQR or Q3 + 1.5 * IQR. NMSS, Non-Motor Symptoms Scale.

Although 291 PD patients (58.2%) presented an increase in the PDQ-39SI score after the 2-year follow-up, only 93 (18.6%) presented a clinically significant HRQoL impairment at V2. Differences in change from V0 to V2 of UPDRS-III, UPDRS-IV, FOGQ, NMSS, BDI-II, PDSS, NPI, VAS-PAIN, VASF-physical, VASF-mental, and ADLS scores between patients with and without clinically significant HRQoL impairment were observed (Table 2). Specifically, PD patients who presented at the 2-year follow-up a clinically significant HRQoL impairment presented a 97.3% increase of the NMS burden (NMSS total score from 29.2 ± 25.87 to 57.84 ± 46.73 [*p* < 0.0001]) compared to 8.6% in those patients who did not (NMSS total score from 48.38 ± 38.59 to 52.53 ± 41.35 [*p* = 0.003]) (Fig. 2A). By domains, the most significant differences were observed for sleep/fatigue (*p* < 0.0001) and mood/apathy (*p* < 0.0001) (Table 2 and Fig. 2B). Moderate correlations were observed between the change from V0 to V2 in the PDQ-39SI score and the score in FOGQ (*r* = 0.34; *p* < 0.0001), NMSS (*r* = 0.41; *p* < 0.0001), BDI-II (*r* = 0.33; *p* < 0.0001) and ADLS (*r* = −0.40; *p* < 0.0001) (Supplementary Table 1).

**Table 2:** Changes in motor and non-motor symptoms and disability in PD patients from V0 (baseline) to V2 (2 years ± 1 month) with regards to presenting or not clinically significant HRQoL impairment.

	Non clinically significant HRQoL impairment *N* = 407	Clinically significant HRQoL impairment *N* = 93	*p*
Age at baseline	63.04 ± 7.99	61.32 ± 10.17	0.354
Gender (males) (%)	60	57	0.341
Disease duration (at V0)	5.65 ± 4.36	4.91 ± 3.55	0.247
Number of non-antipark. drugs (at V0)	2.56 ± 2.36	2.33 ± 2.49	0.220
*Change at V2 (from V0 to V2)*			
LEDD	+177.15 ± 330.2	+228.75 ± 318.27	0.174
Number of non-antipark. drugs	+0.55 ± 1.56	+0.65 ± 1.45	0.685
UPDRS-III (OFF)	+2.25 ± 9.77	+7.76 ± 11.2	**<0.0001**
UPDRS-IV	+0.47 ± 2.47	+1.47 ± 2.55	**0.002**
FOGQ	+0.68 ± 3.85	+3.32 ± 4.71	**<0.0001**
PD-CRS	−2.17 ± 12.18	−0.67 ± 10.12	0.293
NMSS	+4.15 ± 32.03	+28.64 ± 35.65	**<0.0001**
Cardiovascular	+6.21 ± 14.41	+8.11 ± 12.83	0.310
Sleep/fatigue	+0.7 ± 15.96	+12.98 ± 18.42	**<0.0001**
Mood/apathy	+0.5 ± 14.57	+8.62 ± 15.19	**<0.0001**
Perceptual symptoms	+1.89 ± 10.61	+4.35 ± 12.56	0.141
Attention/memory	+1.74 ± 14.16	+7.28 ± 17.30	**0.07**
Gastrointestinal symptoms	+2.19 ± 12.64	+4.9 ± 12.8	**0.020**
Urinary symptoms	+1.29 ± 20.22	+9.28 ± 21.56	**0.001**
Sexual dysfunction	+2.63 ± 30.71	+10.51 ± 23.43	**0.007**
Miscellaneous	+0.72 ± 14.88	+6.19 ± 14.89	**0.011**
BDI-II	−0.63 ± 7.75	+4.51 ± 6.13	**<0.0001**
PDSS	+2.82 ± 25.80	−9.04 ± 24.96	**<0.0001**
QUIP-RS	−0.02 ± 9.25	+0.34 ± 8.06	0.736
NPI	−0.43 ± 4.28	+4.28 ± 8.06	**<0.0001**
VAS-PAIN	+ 0.18 ± 3.21	+1.01 ± 3.74	**0.023**
VASF − physical	+ 0.09 ± 2.97	+1.1 ± 2.92	**0.004**
VASF-mental	−0.12 ± 2.76	+1.05 ± 2.95	**0.002**
ADLS	−2.84 ± 11.08	−10.97 ± 12.42	**<0.0001**

Chi-squared and Mann–Whitney–Wilcoxon test were applied. The results represent percentages or mean ± SD. The symbol “+” indicates an increase in the score of the scale at V2 compared to V0 while the symbol “–” indicates a decrease. Data about UPDRS-III are during the OFF state (first hour in the morning without taking medication in the previous 12 h).

*ADLS* Schwab & England Activities of Daily Living Scale, *BDI-II* Beck Depression Inventory-II, *FOGQ*, Freezing Of Gait Questionnaire, *LEDD* levodopa equivalent daily dose (mg), *NMSS* Non-Motor Symptoms Scale, *NPI* Neuropsychiatric Inventory, *PD-CRS* Parkinson’s Disease Cognitive Rating Scale, *PDSS* Parkinson’s Disease Sleep Scale, *QUIP-RS* Questionnaire for Impulsive-Compulsive Disorders in Parkinson’s Disease-Rating Scale, *UPDRS* Unified Parkinson’s Disease Rating Scale, *VAFS* Visual Analog Fatigue Scale, *VAS-Pain* Visual Analog Scale-Pain.

The bold values indicates statistically significant *p* values.

### Predictors of clinically HRQoL impairment

To be younger (OR = 0.896; 95% CI 0.829–0.968; *p* = 0.006), to be a female (OR = 4.181; 95% CI 1.422–12.290; *p* = 0.009), and to have a greater increase in BDI-II (OR = 1.139; 95% CI 1.053–1.231; *p* = 0.001) and NMSS (OR = 1.052; 95% CI 1.027–1.113; *p* < 0.0001) total scores from V0 to V2 were associated with clinically significant HRQoL impairment at the 2-year follow-up, after adjustment to many covariates (Hosmer–Lemeshow test, *p* = 0.665; *R* ^2^ = 0.655) (Table 3). Specifically, an increase in ≥5 and ≥10 points of BDI-II and NMSS total score at V2 multiplied the probability of presenting a clinically significant HRQoL impairment by 5 (OR = 5.453; 95% CI 1.663–17.876; *p* = 0.005) and 8 (OR = 8.217; 95% CI 2.975–22.696; *p* = 0.002), respectively. When ADLS was included in the model (ADLS at V0 and the change in ADLS score from V0 to V2), only a greater increase in BDI-II (OR = 1.148; 95% CI 1.057–1.258; *p* = 0.001), NMSS (Non-Motor Symptoms Scale) (OR = 1.056; 95% CI 1.029–1.083; *p* < 0.0001) and NPI (OR = 1.072; 95% CI, 1.001–1.147; *p* = 0.046) total scores and a decrease in ADLS score (OR = 0.884; 95% CI 0.820–0.954; *p* < 0.0001) from V0 to V2 were associated with clinically significant HRQoL impairment at the 2-year follow-up (Hosmer–Lemeshow test, *p* = 0.621; *R* ^2^ = 0.718).

**Table 3:** Binary logistic regression model about factors associated with clinically significant HRQoL impairment at V2 (2 years follow-up).

	OR^a^	OR^b^	95% CI^a^	95% CI^b^	*p* ^a^	*p* ^b^
Age	**0.896**	**0.813**	**0.829–0.968**	**0.709–0.933**	**0.006**	**0.003**
Gender (female)	**4.181**	**35.847**	**3.452–372.204**	**1.378–8.424**	**0.009**	**0.003**
Disease duration	1.040	0.734	0.388–1.389	0.763–1.360	0.673	0.342
No. of non-antiparkinsonian drugs/day	0.922	1.005	0.728–1.167	0.697–1.449	0.499	0.979
Change at 2 years follow-up						
LEDD (mg)	1.000	1.002	0.998–1.005	0.998–1.002	0.860	0.385
UPDRS-III	1.056	1.088	0.995–1.120	0.978–1.211	0.071	0.121
UPDRS-IV	0.959	0.756	0.417–1.372	0.650–1.137	0.785	0.358
FOGQ	1.143	1.160	0.906–1.485	1.108–1.559	0.088	0.239
NMSS	**1.052**	**1.069**	**1.027–1.113**	**1.007–1.043**	**<0.0001**	**0.001**
PD-CRS	0.995	0.972	0.900–1.050	0.963–1.045	0.837	0.473
BDI-II	**1.139**	**1.400**	**1.053–1.231**	**1.149–1.705**	**0.001**	**0.001**
NPI	1.037	1.158	0.976–1.103	0.995–1.348	0.238	0.058

Dependent variable: Clinically significant HRQoL impairment (defined as PDQ-39SI_V2_ ≥ 10% PDQ-39SI_V0_). OR and 95% CI are shown. Hosmer–Lemeshow test, *p*_a_ = 0.665; *p*_b_=0.998; *R* ^2^_a_ = 0.655; *R* ^2^_b_ = 745. The model was adjusted to variables at baseline: LEDD (mg), UPDRS-III, UPDRS-IV, FOGQ, NMSS, PD-CRS, BDI-II, NPI, PDQ-39SI.

*BDI-II* Beck Depression Inventory-II, *FOGQ* Freezing Of Gait Questionnaire, *LEDD* levodopa equivalent daily dose (mg), *NMSS* Non-Motor Symptoms Scale, *NPI* Neuropsychiatric Inventory, *PD-CRS* Parkinson’s Disease Cognitive Rating Scale, *PDQ-39SI* 39-item Parkinson’s Disease Quality of Life Questionnaire Summary Index, *QUIP-RS*, Questionnaire for Impulsive-Compulsive Disorders in Parkinson’s Disease-Rating Scale, *UPDRS* Unified Parkinson’s Disease Rating Scale.

^a^All cohort (*n* = 500).

^b^Early PD patients (*n* = 277).

The bold values indicates statistically significant *p* values.

In the subgroup of early PD (*N* = 277), quite similar results, an increase in mean PDQ-39SI from V0 to V2 of 23.4% (from 14.22 ± 11.29 to 17.62 ± 15.36; *p* < 0.0001), were observed. Fifty-six patients (20.2%) presented a clinically significant HRQoL impairment at the 2-year follow-up. However, as in the whole cohort, GQoL did not change significantly (PQ-10, *p* = 0.111; EUROHIS-QOL8, *p* = 0.756). In the binary regression model, as in the all cohort, to be younger (OR = 0.813; 95% CI 0.709–0.933; *p* = 0.003), to be a female (OR = 35.847; 95% CI 3.452–372.204; *p* = 0.003), and to have a greater increase in BDI-II (OR = 1.400; 95% CI 1.149–1.705; *p* = 0.001) and NMSS (OR = 1.069; 95% CI 1.007–1.043; *p* =0.001) total scores from V0 to V2 were associated with clinically significant HRQoL impairment at the 2-year follow-up, after adjustment to many covariates (Hosmer–Lemeshow test, *p* = 0.998; *R* ^2^ = 0.745) (Table 3). When ADLS was included in the model, to be younger (OR = 0.769; 95% CI 0.624–0.946; *p* = 0.013), to be a female (OR = 31.982; 95% CI 1.678–609.587; p = 0.021), and to have a greater increase in BDI-II (OR = 1.197; 95% CI 1.126–1.990; *p* = 0.006), NMSS (Non-Motor Symptoms Scale) (OR = 1.108; 95% CI 1.033–1.188; *p* = 0.004) and NPI (OR = 1.323; 95% CI 1.041–1.681; *p* = 0.022) total scores from V0 to V2 were associated with clinically significant HRQoL impairment at the 2-year follow-up (Hosmer–Lemeshow test, *p* = 0.217; *R* ^2^ = 0.816), but not the change in the ADLS score (OR = 0.889; 95% CI 0.786–1.1006; *p* = 0.062). Moderate correlations were observed between the change from V0 to V2 in the PDQ-39SI score and the score in FOGQ (*r* = 0.39; *p* < 0.0001), NMSS (*r* = 0.41; *p* < 0.0001), NPI (*r* = 0.35; *p* < 0.0001) and ADLS (*r* = −0.41; *p* < 0.0001) (Supplementary Table 2).

### Predictors of the change in the PDQ-39SI from V0 to V2

Finally, similar results were observed in both groups, the whole cohort and the early PD subgroup, when a linear regression model was considered (PDQ-39SI change from V0 to V2 as dependent variable) (Supplementary Table 2). To be a female (*β* = 0.17; *p* < 0.0001) and change in UPDRS-III (*β* = 0.23; *p* < 0.0001), FOGQ (*β* = 0.20; *p* < 0.0001), and NMSS (*β* = 0.37; *p* < 0.0001) scores provided the highest contribution to the model (adjusted R-squared 0.45) in the whole cohort. In early PD patients, the variables associated with HRQoL change at the 2-year follow-up were the same (Supplementary Table 2). When the ADLS score was included in the model, the results were similar but with the ADLS as an independent variable associated with HRQoL change too (*β* = −0.21; 95% CI −0.353, −0.107; *p* < 0.0001; adjusted R-squared 0.45 [*N* = 500; all cohort]; *β* = −0.23; 95% CI −0.433, −0.076; *p* = 0.005; adjusted R-squared 0.431 [*N* = 277; early PD subgroup]).

## Discussion

In this longitudinal follow-up study, we report that there is a significant HRQoL impairment in PD patients in the short-term and that impairment in the motor status during the OFF state (UPDRS-III), increased gait problems (FOGQ), and increased NMS burden contribute to it. Specifically, mood impairment and NMS burden increase were independent factors associated with clinically significant HRQoL impairment at the 2-year follow-up, which one was present in about every 5 patients. Moreover, the results indicate that it will be especially important to be vigilant about clinically significant HRQoL impairment in women and younger patients.

After a 2-year follow-up, PD patients from the COPPADIS cohort demonstrated impairment in motor function (H&Y, UPDRS-III, UPDRS-IV, FOGQ). The increase of motor impairments measured with the UPDRS were in agreement with other studies^27,28^. Also, significant changes in NMS were observed in the NMS burden as a whole, pain, fatigue, and cognition, but not in controls. These results aligned with previous longitudinal studies indicating that the severity of NMS in PD tends to become progressively worse with the course of the disease and also indicate that non-motor evaluation is complementary to measuring PD progression^19,26,29–32^. With respect to the QoL, although more than a half of PD patients presented a PDQ-39SI score at the 2-year follow-up higher than at baseline, only 18.6% presented HRQoL impairment as clinically significant. In a previous study with 707 PD patients followed prospectively for the 2-year as well, 17% worsened clinically while 584 were rated as stable^29^. The results can be vary due to the definition of QoL impairment as clinically significant^31,33,34^. Based on the postal reply of 728 PD patients, Peto et al.^34^ determined that 1.6 points worsening on a PDQ-39SI is the minimal clinically important difference threshold. More recently, Horváth et al.^31^ considered the most optimal estimates threshold for PDQ-39-SI in + 4.22 points for detecting minimal clinically important worsening. However, there is no “gold standard” methodology of estimating the minimal important difference and as the degree of improvement is conditioned by the baseline score; therefore, the use of a percentage might be more appropriate^35,36^. Patients appear to be able to detect changes of 7–10% on QoL instruments or pain scales^36^. In our case, the minimal important difference was considered as an increase of 10% or more in the PDQ-39SI score^33,35–37^. Ten percent of the mean score of the PDQ-39SI in our study represents 1.6 points; therefore, similar to the proposal of Peto et al.^34^. However, in a patient with a higher baseline PDQ-39SI score, for example, 50 points, the minimal clinically significant worsening change should be 5 points. Hence, in less than 1 in 3 patients who had an increase in the PDQ-39 score, this was considered clinically significant. In any case, it seems clear that even in a relatively short follow-up period, patients with PD experience a significant decrease in HRQoL^21,29^. However, as Reuther et al.^22^ reported in 145 PD patients after a 12-month follow-up, there doesn’t seem to be a significant change in QoL generic scales.

For assessing the NMS as a whole, we used the NMSS. To date, this scale has been used in more than 100 clinical studies and trials and it has shown to be capable of detecting longitudinal changes in NMS, where studies have shown differential changes over time of several of the NMSS domains^32,38,39^. Moreover, it has been demonstrated a consistent and strong correlations between NMSS burden and HRQoL measures^32,40–42^. In our study, a very clear difference in the change of NMS burden was observed between patients with and without clinically significant HRQoL impairment. Changes in all domains of the NMSS scale correlated with QoL changes. Similarly, previous studies observed a correlation between NMS burden assessed with the NMSS and QoL changes over time^19^. Moreover, in our analysis, NMS burden progression was an independent factor related to HRQoL impairment. Prakash et al. observed for the first time that non-motor problems provided a better prediction of the change of QoL in 227 PD patients over a 2-year follow-up period^19^. However, they did not provide the variance value of the model, many factors potentially affecting QoL were not included, and what they considered was the baseline NMSS score. On the contrary, in this study we wanted to analyze in detail what changes in many aspects of the disease observed after the 2-year follow-up contributed to a worsening in the patients´ QoL. So, several variables were included, the results of the model represented ~70% of the variance when HRQoL changes were considered, and the changes in all variables were adjusted to the scores at baseline. To our best knowledge, this is the first longitudinal-prospective study analyzing in such detail which are the predictors of QoL impairment in a large sample of PD patients. Reinforcing the idea that the progression of NMS is pivotal to the worsening of the QoL throughout the evolution of the disease, improvements of NMS were associated with improved QoL in advanced parkinsonian patients during 2‐year treatment with levodopa‐carbidopa intestinal gel infusion therapy^43^. In line with this, Erro et al. observed that NMS significantly affected QoL in PD, demonstrating that this was especially the case when patients were in their honeymoon period (during which time the side effects of the disease aren’t too disabling and there is a response to medications)^44^. In the subgroup of early PD patients from our study, the change in the NMSS total score at 2-years was one of the most significant contributors to HRQoL impairment.

Another important factor is mood. Like in other studies, the mean score of BDI didn’t change over time^18,22^, suggesting that depression-type frequency does not appear to change over time in PD^45^. Cross-sectional studies have reported the clear contribution of depression or a worse mood to a poorer QoL in PD patients^3,12,13^. In fact, it was observed in the COPPADIS baseline cross-sectional analysis^14^. However, to our knowledge, this is the first time that mood worsening is identified as an independent factor associated with clinically significant HRQoL impairment in PD patients. This subgroup of patients (N=93) presented a mean increase in the BDI-II score of 4.5 points at the 2-year follow-up and specifically, an increase in ≥5 points multiplied by 5 the probability of presenting a clinically significant HRQoL impairment, independent of other factors. Reuther et al.^22^ identified depression as the strongest predictor for reduced HRQoL in 145 PD patients after 1-year follow-up. However, we identified the change in the score of the BDI-II as a predictor of clinically significant HRQoL impairment after adjustment to BDI-II score at baseline. From a practical point of view, our findings suggest an important role of the neurologist being alert to a possible worsening of mood, as well as greater NMS burden, in patients with PD throughout the evolution of the disease since this is what impacts on the patient’s QoL. Knowing what impacts on the QoL and contributes to its worsening, depending on the variable, intervention measures with the intention of correcting them can be proposed^46^. Studies demonstrating a QoL improvement correlated with mood and NMS burden improvement have been published^47^. With regards of the results observed here, it should be necessary to be alert about mood and NMS burden changes over time, especially in younger patients and females. A mildly significant gender difference in disability and QoL reporting has been noted, with women citing greater disability and reduced QoL^48,49^. Depression and fatigue were the major causes of low HRQoL in women even in the early phases of PD^50^. To attenuate this sex difference in disease experience, psychological distress screening and management (particularly targeting females) should be considered as part of PD clinical care^23^. Moreover, QoL, as measured on the PDQ-39, is significantly worse in young-onset PD patients than in older-onset PD patients, and young-onset PD patients also experience loss of employment, disruption of family life, greater perceived stigmatization, and depression than do older-onset PD patients^51,52^.

The most important limitation of this study is the fact that information about follow-up was recorded only in 524 patients of 695 initially included in the study (75.5%). Of them, data for the PDQ-39, PQ-10, and EUROHIS-QOL8 at baseline and at V2 was available in 500, 503, and 507 PD patients, respectively. Thirty-eight patients (5.5%) dropped out of the study (1 death; 2 with change in diagnosis; 35 other reasons) at the 2-year follow-up and 132 (19%) were not assessed. However, this is a limitation observed in other prospective studies. Of 7507 PD patients, follow-up data was available only for 4680 participants (62.3%)^53^. In the study of Antonini et al.^29^, 707 PD patients from 1142 initially included (61.9%) were evaluable at 24 months. An important second limitation is that PD patients older than 75 years old were excluded from participation by COPPADIS study protocol^14^, which leads to an early PD bias in this cohort. For some variables, the information was not collected in all cases. Moreover, this is a multicenter mono-country study, being the ideal for this type of studies the participation of patients from different parts of the world, so the results should be considered with caution when extrapolating them to the general PD population (i.e., race, country healthcare, etc.). By the contrary, strengths of our study include a very complete assessment, the large sample size, a prospective longitudinal follow-up design, the fact that this analysis was “a priori” planned as one objective of the multicenter COPPADIS project^16^, and the extensive clinical and demographic information recorded.

The findings of this study have important implications in daily clinical practice. In a disorder like PD in which one there is no a cure, treatment is symptomatic and the aim is to improve the patient’s QoL. This is complex because many factors influence QoL in PD. Furthermore, PD is a complex disorder with many manifestations and with a great variability in its progression among patients. Regarding this study observations, some important points should be considered in daily clinical practice. First, a complete assessment of the patient with PD periodically including motor status, NMS, QoL and disability should be the ideal practice. Second, NMS progression contributes significantly to a QoL worsening and it is crucial its evaluation. Very interestingly, we reported very recently that PD patients from the COPPADIS cohort with a lower H&Y stage but a greater global NMS burden may have a worse QoL than patients with a higher H&Y stage but lower global NMS^54^. Third, mood is another key factor to consider whenever we evaluate the patient in clinical practice. Fourth, we have to keep in mind that mood impairment and global NMS progression predict a patient´s QoL worsening. Finally, we should be especially careful in all of the above in the case of a female patient and in young patients.

A problem in clinical practice is the lack of time to evaluate the patient. For the PD patient, to bring adequately covered questionnaires to the consultation, for example with the help of nursing staff, or even in the future with mobile applications that transfer the data to the patient’s medical record, it could be a possibility that facilitates the complete and comprehensive assessment. In general, it is something that is not done today, and proof of this is the alarming lack of literature about the global progression of the disease including NMS in large cohorts of patients. More studies with large PD cohorts and long-term follow-up are required. Our aim with the COPPADIS cohort is to follow for 5 years^55^. Collecting data from different cohorts and making comparisons would also be of great interest.

In conclusion, the present study observes HRQoL impairment in PD patients in a short 2-year follow-up, even in early PD patients, but not the GQoL. A younger age, to be a female, and mood and NMS burden impairment were associated with clinically significant HRQoL impairment after the 2-year follow-up. The progression of NMS is pivotal in the worsening of the QoL throughout the evolution of the disease in PD, and it is necessary to keep in mind to ask for mood or NMS changes, especially in females and young patients.

## Methods

PD patients and controls who were recruited from January 2016 to November 2017 (baseline visit; V0) and evaluated again at the 2-year follow-up (V2) from 35 centers of Spain from the COPPADIS cohort^56^, were included in the study. Methodology about COPPADIS-2015 has been previously published^57^. This is a multicenter, observational, longitudinal-prospective, 5-year follow-up study designed for analyzing disease progression in a Spanish population of PD patients. Specifically, 17 objectives were proposed in the protocol^55^. Even though the recruitment period ended in October 2017, the prospective follow-up phase is ongoing. Patients, caregivers (patient´s primary caregiver), and controls (subjects without PD and any other severe and disabling concomitant disorder) were included^55^. Annual visits from V0 (baseline) to V5 (60 moths ± 3 months) are conducted to the patients and at V0, V2, V4, and V5 to the controls and caregivers. All patients included were diagnosed according to UK PD Brain Bank criteria^57^. Exclusion criteria^55^ were: non-PD parkinsonism, dementia criteria (Mini Mental State Examination [MMSE] ≥ 26), age < 18 or >75 years, inability to read or understand the questionnaires, to be receiving any advanced therapy (continuous infusion of levodopa or apomorphine, and/or with deep brain stimulation), and presence of comorbidity, sequelae, or any disorder that could interfere with the assessment.

Information on sociodemographic aspects, factors related to PD, comorbidity, and treatment were collected. V0 and V2 evaluations included^55^: (1) motor assessment (Hoenh & Yahr [H&Y]^58^, Unified Parkinson’s Disease Rating Scale [UPDRS] part III and part IV^59^, Freezing of Gait Questionnaire [FOGQ]^60^; (2) NMS (Non-Motor Symptoms Scale [NMSS]^61^, Parkinson’s Disease Sleep Scale [PDSS]^62^, Visual Analog Scale-Pain [VAS-Pain]^63^, Visual Analog Fatigue Scale [VAFS]^64^, cognition (MMSE^65^, Parkinson’s Disease Cognitive Rating Scale [PD-CRS]^66^, completing a simple 16-piece puzzle); (3) mood and neuropsychiatric symptoms (Beck Depression Inventory-II [BDI-II]^67^, Neuropsychiatric Inventory [NPI]^68^, Questionnaire for Impulsive-Compulsive Disorders in Parkinson’s Disease-Rating Scale [QUIP-RS]^69^; (4) and disability (Schwab & England Activities of Daily Living Scale [ADLS]^70^. In patients with motor fluctuations, the motor assessment was made during the OFF state (without medication in the last 12 h) and during the ON state. On the other hand, the assessment was only conducted without medication in patients without motor fluctuations. The same evaluation as for the patients, except for the motor assessment, was conducted in control subjects at V0 and at V2 (2 years ± 1 month). Three scales were used to assess QoL at V0 and at V2^28^: (1) the 39-item Parkinson’s disease Questionnaire (PDQ-39)^71^, (2) a rating of global perceived QoL (PQ-10) on a scale from 0 (worst) to 10 (best)^13^, and (3) the EUROHIS-QOL 8-item index (EUROHIS-QOL8)^72^. The PDQ-39 is a PD-specific questionnaire that assesses the patients’ HRQoL. There are 39 items grouped into 8 domains: (1) Mobility (items 1 to 10); (2) Activities of daily living (items 11 to 16); (3) Emotional well-being (items 17 to 22); (4) Stigma (items 23 to 26); (5) Social support (items 27 to 29); (6) Cognition (items 30 to 33); (7) Communication (items 34 to 36); (8) Pain and discomfort (items 37 to 39). For each item, the score may range from 0 (never) to 4 (always). The symptoms refer to the 4 weeks prior to assessment. Domain total scores are expressed as a percentage of the corresponding maximum possible score and a Summary Index is obtained as average of the domain scores. The EUROHIS-QOL8 is an 8-item GQoL questionnaire (quality of life, health status, energy, autonomy for activities of daily living, self-esteem, social relationships, economic capacity, and habitat) derived from the WHOQOL-BREF. For each item, the score ranges from 0 (not at all) to 5 (completely). The total score is expressed as the mean of the individual scores. A higher score indicates a better QoL. In controls, only the PQ-10 and the EUROHIS-QOL8 were assessed.

Clinically significant HRQoL impairment was defined as presenting an increase in PDQ-39SI score at V2 ≥ 10% of score at baseline (V0) whereas GQoL impairment as presenting a decrement in PQ-10 and/or EUROHIS-QOL8 score at V2 ≤ 10% of score at baseline (V0)^33^. Taking into account that in the COPPADIS cohort the range of disease duration varies from <1 year to 30 years and based on the general response to treatment and progression of symptoms in PD and considering a recent publication of this same cohort^73^, patients with ≤5 years of disease duration were considered as early PD patients.

### Data analysis

Data were processed using SPSS 20.0 for Windows. For comparisons between patients and controls, the Student’s *t*-test, Mann–Whitney *U* test, Chi-square test, or Fisher test were used as appropriate (distribution for variables was verified by one-sample Kolmogorov–Smirnov test). The Wilcoxon-signed rank test was performed to test whether the mean differences of the PDQ-39SI, PQ-10, and EUROHIS-QOL8 scores and the individual PDQ-39SI and EUROHIS-QOL8 domain scores between the two visits (V0 and V2) were significant. This test and/or the marginal homogeneity test were applied for other scales for analyzing the change from V0 to V2. Spearman’s or Pearson’s correlation coefficient, as appropriate, were used for analyzing the relationship between continuous variables. Correlations were considered weak for coefficient values ≤0.29, moderate for values between 0.30 and 0.59, and strong for values ≥0.60.

Clinically significant QoL impairment was expressed as a percentage and it was only calculated if the change between scores (PDQ-39SI; PQ-10; EUROHIS-QOL8) from V0 to V2 was significant. For determining predictive factors of QoL impairment, a logistic regression model (QoL impairment as dependent variable) was performed. The model was well-planned, as recommended by best-practice methods^74^, in which known and presumably predictor variables affecting QoL changes (dependent variable) were included: change from V0 to V2 in levodopa equivalent daily dose (LEDD)^75^, UPDRS-III-OFF (motor severity), UPDRS-IV (motor complications), FOGQ, NMSS (NMS burden), PD-CRS (cognition), BDI-II (mood), and NPI (neuropsychiatric symptoms). The model was adjusted to baseline QoL and age, gender, disease duration, comorbidity (total number of non-antiparkinsonian medications as surrogate marker^14^), and the score of the rest of the variables at baseline (LEDD, UPDRS-III-OFF, UPDRS-IV, FOGQ, NMSS, PD-CRS, and NPI). Disability (ADLS) was not included in the model because this is consequence of symptoms, but since it is related to QoL, in a second model ADLS at baseline and change in ADLS from V0 to V2 were included. Hosmer–Lemeshow test was applied and adjusted R-squared was calculated for all analyses. Finally, multiple linear regressions were performed with “change in QoL” as dependent variable but only for variables (PDQ-39SI; PQ-10; EUROHIS-QOL8) changing significantly from V0 to V2. The independent variables included were the same as in the binary model. The p-value was considered significant when it was <0.05.

### Standard protocol approvals, registrations, and patient consents

The Comité de Ética de la Investigación Clínica de Galicia from Spain (2014/534; 02/DEC/2014) approval was obtained. Written informed consents from all participants in this study were obtained before the start of the study. COPPADIS-2015 was classified by the AEMPS as a Post-authorization Prospective Follow-up study with the code COH-PAK-2014-01.

### Reporting summary

Further information on research design is available in the Nature Research Reporting Summary linked to this article.

## Supplementary information

Reporting Summary

Supplementary Information

## Data Availability

The data that support the findings of this study are available from the corresponding author upon reasonable request.

## Code availability

No computer coding was used in the completion of the current manuscript.
