# Supplementary material for: Predictors of clinically significant quality of life impairment in Parkinson’s disease
Source: NPJ Parkinsons Dis. 2021 Dec 16;7:118. doi: 10.1038/s41531-021-00256-w (PMC8677846; doi:10.1038/s41531-021-00256-w)
Supplement: Supplementary file 2 — Supplementary Information [file 41531_2021_256_MOESM2_ESM.pdf]

Supplementary Table 1. Correlations between the change from V0 to V2 in the PDQ-39SI score and the change in other variables.

	PDQ-39SI All cohort N=500	p	PDQ-39SI Early PD group N=277	p
Age at baseline	-0.025	0.573	-0.071	0.241
Disease duration (at V0)	0.036	0.435	0.030	0.614
Number of non-antipark. drugs (at V0)	-0.030	0.499	-0.026	0.670
**Change at V2 (from V0 to V2)**				
LEDD	0.040	0.382	0.113	0.068
Number of non-antipark. drugs	0.004	0.936	0.016	0.800
UPDRS-III (OFF)	0.289	**<0.0001**	0.288	**<0.0001**
UPDRS-IV	0.196	**<0.0001**	0.219	**<0.0001**
FOGQ	0.343	**<0.0001**	0.396	**<0.0001**
PD-CRS	0.040	0.377	0.080	0.192
NMSS	0.417	**<0.0001**	0.414	**<0.0001**
- Cardiovascular	0.092	**0.042**	0.124	**0.040**
- Sleep / fatigue	0.340	**<0.0001**	0.306	**<0.0001**
- Mood / apathy	0.358	**<0.0001**	0.367	**<0.0001**
- Perceptual symptoms	0.150	**0.001**	0.100	0.069
- Attention / memory	0.233	**<0.0001**	0.217	**<0.0001**
- Gastrointestinal symptoms	0.203	**<0.0001**	0.212	**<0.0001**
- Urinary symptoms	0.189	**<0.0001**	0.162	**0.007**
- Sexual dysfunction	0.123	**0.006**	0.173	**0.004**
- Miscellaneous	0.170	**<0.0001**	0.242	**<0.0001**
BDI-II	0.331	**<0.0001**	0.299	**<0.0001**
PDSS	-0.247	**<0.0001**	-0.295	**<0.0001**
QUIP-RS	0.117	**0.014**	0.096	0.139
NPI	0.290	**<0.0001**	0.358	**<0.0001**
VAS-PAIN	0.207	**<0.0001**	0.262	**<0.0001**
VASF − physical	0.226	**<0.0001**	0.227	**<0.0001**
VASF – mental	0.248	**<0.0001**	0.293	**<0.0001**
ADLS	-0.407	**<0.0001**	-0.411	**<0.0001**

Spearman’s rank correlation coefficient was applied.

ADLS, Schwab & England Activities of Daily Living Scale; BDI-II, Beck Depression Inventory-II; FOGQ, Freezing Of Gait Questionnaire; LEDD, levodopa equivalent daily dose (mg); NMSS, Non-Motor Symptoms Scale; NPI, Neuropsychiatric Inventory; PD-CRS, Parkinson’s Disease Cognitive Rating Scale; PDSS, Parkinson’s Disease Sleep Scale; QUIP-RS, Questionnaire for Impulsive-Compulsive Disorders in Parkinson’s Disease-Rating Scale; UPDRS, Unified Parkinson’s Disease Rating Scale; VAFS, Visual Analog Fatigue Scale; VAS-Pain, Visual Analog Scale-Pain.

Supplementary Table 2. Multiple linear regression model for PDQ-39SI change from V0 to V2 as dependent variables.

	Adjusted R-squared	β standardized coefficient	CI 95%	*P* value
**All cohort (N=500)**	**0.450**			
Gender (female)		0.171	2.069, 6.716	<0.0001
Change in UPDRS-III (OFF)		0.236	0.148, 0.413	<0.0001
Change in FOGQ		0.204	0.308, 0.989	<0.0001
Change in NMSS		0.372	0.096, 0.176	<0.0001
**Early PD patients (N=277)**	**0.408**			
Gender		0.156	0.739, 6.088	0.013
Change in UPDRS-III (OFF)		0.207	0.034, 0.383	0.019
Change in FOGQ		0.293	0.366, 1.196	<0.0001
Change in NMSS		0.312	0.046, 0.147	<0.0001

Dependent variable: HRQoL change from V0 to V2. β standardized coefficient and 95% IC are shown. The model was adjusted to variables at baseline (LEDD, number of non-antiparkinsonian drugs, UPDRS-III, UPDRS-IV, FOGQ, NMSS, PD-CRS, BDI-II, NPI, PDQ-39SI) and changes from Vo to V2 (LEDD, UPDRS-III, UPDRS-IV, FOGQ, NMSS, PD-CRS, BDI-II, NPI). The results includes those variables analysing the change from V0 to V2 with significant impact ($p<0.05$) on dependent variable.

FOGQ, Freezing Of Gait Questionnaire; NMSS, Non-Motor Symptoms Scale; UPDRS, Unified Parkinson's Disease Rating Scale.
